# Neuroinflammation in the NTS is associated with changes in cardiovascular reflexes during systemic inflammation

**DOI:** 10.1186/s12974-019-1512-6

**Published:** 2019-06-20

**Authors:** Mateus R. Amorim, Júnia L. de Deus, Rafael A. Cazuza, Clarissa M. D. Mota, Luiz E. V. da Silva, Gabriela S. Borges, Marcelo E. Batalhão, Evelin C. Cárnio, Luiz G. S. Branco

**Affiliations:** 1 Dental School of Ribeirão Preto, University of São Paulo, Ribeirão Preto, SP 14040-904 Brazil; 2 Ribeirão Preto Medical School, University of São Paulo, Ribeirão Preto, SP 14049-900 Brazil; 3 School of Philosophy, Science and Literature of Ribeirão Preto, University of São Paulo, Ribeirão Preto, SP 14040-901 Brazil; 4 Nursing School of Ribeirão Preto, University of São Paulo, Ribeirão Preto, SP 14040-902 Brazil

**Keywords:** Baroreflex, Chemoreflex, Bezold-Jarisch reflex, Blood pressure, Microglia, Endotoxin

## Abstract

**Background:**

Lipopolysaccharide (LPS)-induced systemic inflammation (SI) is associated with neuroinflammation in the brain, hypotension, tachycardia, and multiple organs dysfunctions. Considering that during SI these important cardiovascular and inflammatory changes take place, we measured the sensitivity of the cardiovascular reflexes baroreflex, chemoreflex, and Bezold-Jarisch that are key regulators of hemodynamic function. We also evaluated neuroinflammation in the nucleus tractus solitarius (NTS), the first synaptic station that integrates peripheral signals arising from the cardiovascular and inflammatory status.

**Methods:**

We combined cardiovascular recordings, immunofluorescence, and assays of inflammatory markers in male Wistar rats that receive iv administration of LPS (1.5 or 2.5 mg kg^−1^) to investigate putative interactions of the neuroinflammation in the NTS and in the anteroventral preoptic region of the hypothalamus (AVPO) with the short-term regulation of blood pressure and heart rate.

**Results:**

LPS induced hypotension, tachycardia, autonomic disbalance, hypothermia followed by fever, and reduction in spontaneous baroreflex gain. On the other hand, during SI, the bradycardic component of Bezold-Jarisch and chemoreflex activation was increased. These changes were associated with a higher number of activated microglia and interleukin (IL)-1β levels in the NTS.

**Conclusions:**

The present data are consistent with the notion that during SI and neuroinflammation in the NTS, rats have a reduced baroreflex gain, combined with an enhancement of the bradycardic component of Bezold-Jarisch and chemoreflex despite the important cardiovascular impairments (hypotension and tachycardia). These changes in the cardiac component of Bezold-Jarisch and chemoreflex may be beneficial during SI and indicate that the improvement of theses reflexes responsiveness though specific nerve stimulations may be useful in the management of sepsis.

## Background

Acute activation of innate immunity during infection or injury is an evolutionary trend protecting organisms against threats [1]. However, exacerbated release of pro-inflammatory mediators, observed during systemic inflammation (SI), causes severe tissue hypoperfusion contributing to multiple organ dysfunction in septic patients [2–4]. Despite recent therapeutic trials for the treatment of sepsis using activated protein C, early goal-directed therapy, and low-dose hydrocortisone, little progress has been made in reducing sepsis mortality [5]. On the other hand, the small number of sepsis survivors exhibit poor prognoses due to a myriad of pathological events [6–9]. Therefore, it is essential to investigate the acute and long-term cardiovascular complications after SI.

Used as the most accepted experimental model of SI, bacterial compounds, such as the lipopolysaccharide-(LPS), have emerged as the major source of the cellular and molecular cascade associated with systemic inflammatory responses without the infectious component [10–15]. LPS activates mast cells and macrophages receptors causing synthesis and release of relatively high amounts of pro-inflammatory cytokines which lead to vascular damage, hypotension, tachycardia, hypothermia followed by fever, and potentially death [4, 16–18].

Recent evidence indicates that the nervous system regulates immune responses, and vice-versa. This interaction is further strengthened in SI [1, 19]. As to the afferent pathways, vagal afferent fibers do express cytokine receptors, but pro-inflammatory cytokines fail to affect vagus nerve activity [20]. Inflammatory signals convey from different structures such as carotid bodies [3] to the dorsal vagal complex located in the brainstem that encompasses the nucleus tractus solitarius (NTS) with projections to other brainstem and forebrain areas [21]. In the brain, resident microglia are the first line of defense involved in immune responses, that when activated alter their morphology and secrete pro-inflammatory cytokines [22]. After integrating systemic information, the brain modulates the immune system via the recruitment of the autonomic nervous system [23] which includes the efferent arc of the inflammatory reflex that innervate the spleen [1, 24]. These important reflexes controlling inflammation have been documented to be relevant in joint inflammation, rheumatoid arthritis, cardiovascular diseases, and SI [1, 19, 25–27].

Baroreflex, Bezold-Jarisch, and chemoreflex play a pivotal role in homeostatic regulation of arterial pressure and heart rate by establishing synaptic contacts in the NTS [28, 29] and ultimately promoting autonomic adjustments in the heart and resistance vessels [30]. Electrical activation of the carotid sinus nerve and baroreflex afferents attenuates inflammation by unknown mechanisms [25, 31]. Thus, we characterized putative changes in all the cardiovascular reflexes during SI, besides assessing inflammatory markers in the plasma and NTS. Interleukin (IL)-1β was evaluated based on the fact that LPS administration induces microglial activation and enhancement in this pro-inflammatory cytokine in the brain [32] and more specifically in the medulla oblongata [33]. Concomitant with IL-1β surges, IL-10 was evaluated in plasma and NTS as an important anti-inflammatory cytokine that downregulates microglial activation [34]. We hypothesized herein that these cardiovascular reflexes are active and lessening the LPS-induced alterations in vital cardiovascular parameters (hypotension, tachycardia, and autonomic regulation).

## Methods

### Animals

A total of 58 7- to 8-week-old male Wistar rats (300–350 g) from the Animal Care Facility of the University of São Paulo at Ribeirão Preto were used in this study. All the experimental protocols were approved by The Ethics Committee on Animal Research of the Dental School of Ribeirão Preto - University of São Paulo, Ribeirão Preto, Brazil (#2017.1.585.58.9). Rats were kept four per cage in the animal facility of the Dental School of Ribeirão Preto, University of São Paulo under standard daily 12 h light/12 h dark cycle (06:00–18:00), controlled room temperature (23–24 °C), and access to water and chow ad libitum. Taking into account the 3R principles (replacement, reduction, and refinement) as well as the ethical principles that our Ethics Committee operates, we reduced the number of rats of the present study by using only one group that received saline in most of the experimental procedures. It was possible, given that no significant changes in the cardiovascular, inflammatory, and thermoregulatory responses are observed after this vehicle administration.

### Drugs

*Escherichia coli* LPS [(1.5 or 2.5 mg kg^−1^, 0111: B4 dissolved in pyrogenic-free saline [35]], potassium cyanide [KCN, 40 μg diluted in 0.05 mL of saline (Sal) [36]], and phenylbiguanide [(PBG, 5.0 μg kg^−1^) [37]] were purchased from Sigma-Aldrich, USA.

### Surgeries for arterial, venous catheterization, and datalogger implantation

Rats were deeply anesthetized with ip injection of a mixture of ketamine (100 mg kg^−1^) and xylazine (10 mg kg^−1^) and after the absence of reflex response of withdrawal reflex to paw and tail pinching, they were implanted with polyethylene catheters (PE-10 connected to PE-50 tubing; Clay Adams, Parsippany, NJ, USA, Intramedic, Becton Dickinson, Sparks, MD, EUA), into the abdominal aorta via femoral artery and vein for arterial pressure and heart rate recordings and drug administration, respectively. The distal ends of the catheters were tunneled subcutaneously to the back of the neck. In the same surgical procedure, a previously programmed datalogger capsule (SubCue, Calgary, AB, Canada) was inserted into the abdominal cavity of rats through a median laparotomy to record deep body temperature (Tb) at each 5 min. This surgical procedure was carried out in aseptic conditions and additional doses of analgesic was given if any sign of pain was observed. Rats were kept in individual cages and allowed to recover for 24 h at 24 °C before the cardiovascular recordings, that was carried out in conscious freely moving rats. This recovery period was chosen based on previous studies showing that the evaluation of cardiovascular reflexes and inflammatory responses lead to consistent and reproducible results [18, 38].

### Physiological experiments in freely behaving rats

On the day after surgery for blood vessels catheterization, the arterial catheter was connected to a pressure transducer (MLT0380; ADInstruments) that was connected to an amplifier (Bridge Amp, ML221; ADInstruments). The cardiovascular signals were recorded using the Chart Pro software (ADInstruments). After initial adaptation, conscious freely moving rats had the pulsatile arterial pressure (PAP) and heart rate (HR) recorded during 30 min (min) under baseline conditions and throughout 180 min after LPS or Sal administration. The venous catheter was connected to a polyethylene extension for infusion of drugs. Dataloggers capsules were programmed to record Tb at each 5 min for 1 h after and throughout 24 after LPS or Sal administration and the data were applied and calibrated using the SubCue software (SubCue, Calgary, AB, Canada).

#### Spontaneous baroreflex function and spectral analysis

Beat-by-beat series of pulse interval (PI) and systolic arterial pressure (SAP) were derived from the raw PAP recordings and cardiovascular variability was assessed using the open access software CardioSeries [39]. The spontaneous baroreflex function was evaluated by the sequence technique [40, 41]. In a nutshell, the method searches for ramps of SAP values (up or down), lengthening at least 3 points, which are followed by a ramp of PI values in the same direction (up or down) and with the same length. Only SAP ramps with at least 1 mmHg of change between values were considered. Moreover, we assumed a delay of three beats between SAP and PI ramps, which was chosen based on pilot experiments, as well as on previous studies documenting consistent and reproducible results [42]. One baroreflex sequence consists of the consecutive pairs (SAP, PI) that satisfy the abovementioned criteria. Sequences with a linear correlation between SAP and PI greater than 0.8 had their slope calculated and the mean slope over all sequences was taken as the spontaneous baroreflex sensitivity (BRS). In addition, the baroreflex effectiveness index (BEI) was calculated as the number of baroreflex sequences divided by the overall number of SAP ramps, representing the proportion of SAP ramps that effectively produced a reflex response to PI [43].

To investigate eventual acute and long-lasting autonomic imbalance during SI, spectral analysis parameter settings using beat-by-beat series of SAP and PI were analyzed by using the software CardioSeries v2. The analyses were performed in the frequency domain in which the power spectra of PI and SAP were estimated by sequential sets of 512 points (Welch periodogram). Segments containing artifacts or transients that could affect the calculation of power spectral were excluded. The PI spectra were integrated in two frequency bands, i.e., low (LF; 0.2–0.75 Hz) and high-frequency (HF, 0.75–3 Hz). The power of the obtained oscillatory components was quantified in LF band normalized units represented by LF/(LF + HF), whereas the power of the HF band was evaluated in absolute units, since these LF and HF bands representations provide the best correlation of spectral indices of the sympathetic and parasympathetic modulation of the heart rate, respectively. SAP spectra were integrated at LF band only. Data from very low frequency (VLF, 0.00–0.20 Hz) was not used in the present study.

### Plasma measurements of IL-1β and IL-10

At the end of the cardiovascular recordings, arterial blood was withdrawn in EDTA-coated tubes and centrifuged (20 min at 3500 rpm, 4 °C), for plasma extraction, on the third or twenty-fourth hours after Sal or LPS administration. All plasma samples were immediately kept at − 80 °C until assays. Plasma samples were assayed for measurement of the pro-inflammatory cytokine interleukin (IL)-1β (detection limits of 62.5–4000 pg mL^−1^) and the anti-inflammatory cytokine IL-10 (detection limits of 62.5–4000 pg mL^−1^, R & D Systems. Minnesota, USA) using enzyme-linked immunosorbent assay (ELISA) kits according to standard instructions.

### Immunofluorescence

After cardiovascular recordings, rats were deeply anesthetized with ketamine and xylazine and perfused transcardially with ice-cold phosphate-buffered saline (PBS, 0.01 M, 150 mL at pH 7.4) followed by an ice-cold paraformaldehyde (PFA, 350 mL) solution. Afterward, whole brains were removed and postfixed in 4% PFA for 3 h at 4 °C. Fixed brains were cryoprotected at 4 °C with 0.01 M PBS containing 30% sucrose for 72 h. Free-floating sections (30 μm) containing the frozen NTS region were obtained with a cryostat Leica and then stored in a cryoprotectant solution at − 20 °C before further processing. Sections were blocked with 10% normal goat serum (1 h) and then incubated for 24 h in a cocktail of primary antibodies for the labeling of ionized calcium binding adaptor molecule-1 (Iba-1, WAKO, SAR6502 1:1000) or glial fibrillary acidic protein (GFAP, Ab5804, Millipore, Massachusetts, USA 1:5000). Reactions with primary antibodies were followed by 4 h of incubation in the presence of a fluorescent-labeled secondary antibody (Alexa Fluor 488 Abcam, Cambridge, UK, 1:1000). Finally, the sections were mounted on gelatin-coated slides and covered with mounting medium with DAPI, for nuclear staining of all cells present in the slice [44]. Images from the different experimental groups were captured together in parallel using the AxiosKop2 (Carl Zeiss, Germany) optical microscope and an AxioCam Hrc (Carl Zeiss) digital camera, connected to a computer, equipped with Axio Vision 3.1 (Carl Zeiss).

Maximum intensity projections of the images containing NTS region were exported to the ImageJ software (version 1.49v-National Institute of Health, USA), processed in an identical way to reduce the background signals and used to quantify the overall number of microglia. Quantitative analysis of images counting of Iba-1 labeled microglia of NTS was counted using ImageJ software by means of the identification of the central canal and area postrema [45]. A rectangular region containing NTS bilaterally was selected for each individual image and its size was the same for the every slices image [46].

All immunofluorescent analyzes (number, morphology of microglia and GFAP intensity) were carried out by an examiner who did not know the identification of the groups using the ImageJ software and plugins for counting morphological analyzes. For each animal, the means for three slices of the commissural and intermediate NTS were calculated.

### NTS and anteroventral preoptic region of the hypothalamus sample collection for IL-1β and IL-10 assays

**Fig. 1:**
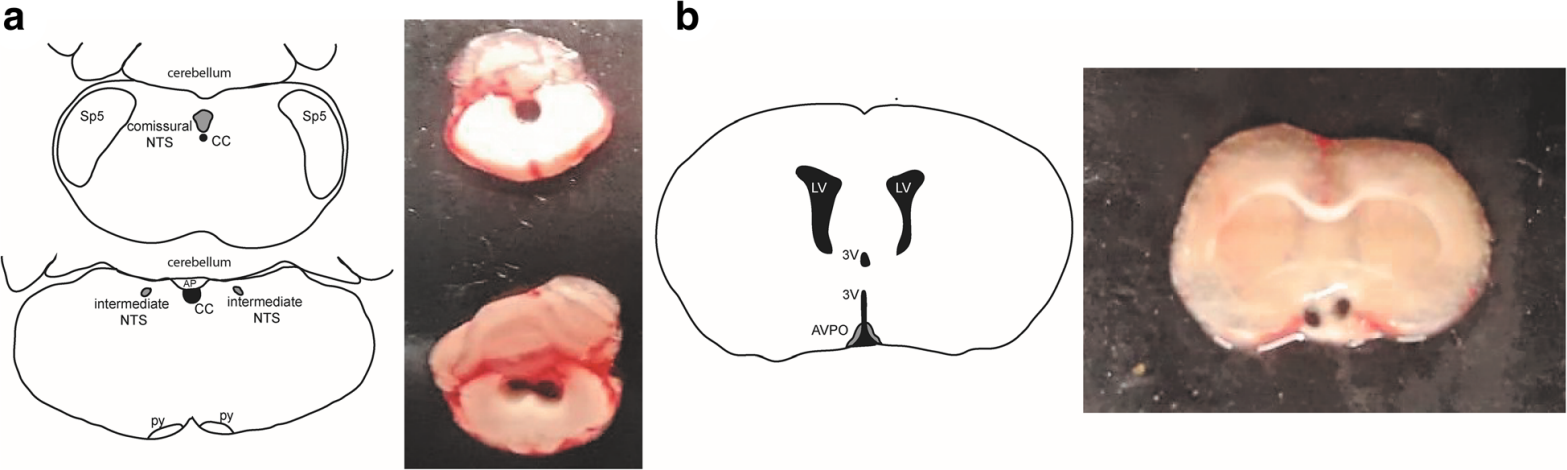
Schematic drawing and representative photo of a punch in the NTS (**a**) and AVPO (**b**). Central canal (cc); pyramidal tract (py); spinal trigeminal nucleus (Sp5); area postrema (AP); nucleus tractus solitarius (NTS); lateral ventricle (LV); third ventricle (3 V); anteroventral preoptic region of the hypothalamus (AVPO)

After recordings, rats were decapitated and the brains were carefully collected, frozen in dry-ice-cold isopentane and stored at − 80 °C until assays for IL-1β and IL-10. Using a cryostat, slices of 1200 μm thickness were prepared to obtain punches of the NTS (Fig. 1a). Bilateral anteroventral preoptic region of the hypothalamus (AVPO) samples were obtained with a cryostat from 1200 μm thick and a punch needle. Punches was done based on the following anatomical references ventral, optic chiasm; dorsal, anterior commissure; median, the third ventricle (Fig. 1b). Three punches from each rat were pooled. Samples were thawed and homogenized in 200 μL of PBS and protease inhibitor cocktail (Cell Signaling, Massachusetts, USA) and then centrifuged at 13,000 rpm for 20 min at 4 °C. Tissue supernatant samples were used to measure IL-1β (detection limits 18.5–4500 pg mL^−1^) and IL-10 levels (detection limits 19.7–4790 pg mL^−1^) by a multiplex assay (LXSARM - 05, R&D System, Minnesota, USA) with Luminex® Magpix™ technology (Austin, TX, USA). Results from IL-1β and IL-10 in NTS and AVPO homogenates were normalized by protein concentrations, which were assessed by the Bio-Rad protein assay based on the Bradford assay (#5000205, Bio-Rad Laboratories, USA).

### Experimental procedures

All the experiments were done at 3 and 24 h after iv administration of Sal or LPS.

1. PAP and HR were recorded throughout 3 and after 24 h. Mean arterial pressure (MAP), HR, spectral analysis of PI and SAP, spontaneous baroreflex gain, and Tb were evaluated offline.
2. Rats were deeply anesthetized with ketamine and xylazine and perfused transcardially with PBS and PFA solution. Afterwards, slices containing NTS were stained for Iba-1 or GFAP.
3. Arterial plasma was withdrawn and rats were decapitated and brains were collected for IL-1β and IL-10 assays in plasma, NTS, and AVPO.
4. During cardiovascular recordings, peripheral chemoreceptors were activated by iv bolus administration of KCN at 3 and 24 h after Sal or LPS administration. KCN was not given until the MAP and HR was stable.
5. During cardiovascular recordings, the Bezold–Jarisch reflex was activated by iv bolus administration of PBG at 3 and 24 h after Sal or LPS administration. PBG was not given until the MAP and HR was stable.

### Statistical analysis

Data are presented as means ± SEM. For comparisons of multiple groups, one-way or two-way ANOVA followed by the Bonferroni post-test were used. Significant differences between groups were considered when *P* ≤ 0.05.

## Results

**Fig. 2:**
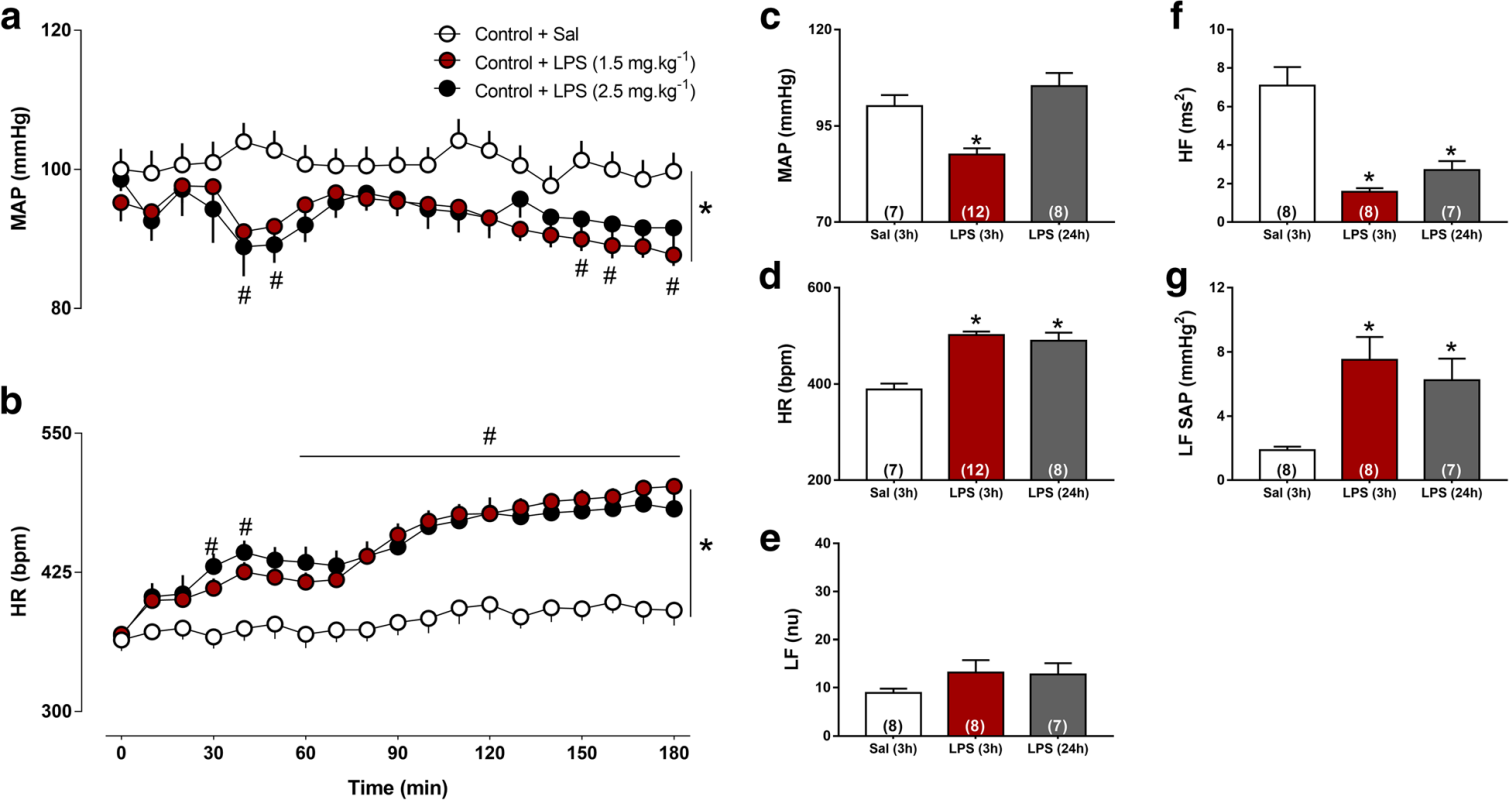
Mean arterial pressure (MAP, **a**) and heart rate (HR, **b**) were continuously monitored throughout 180 min in conscious rats that received iv administration of saline (Sal, *n* = 12) or LPS [1.5 (*n* = 12) and 2.5 mg kg^−1^(*n* = 07)]. Note the acute hypotension and tachycardia after LPS administration. MAP (**c**) and HR (**d**) at 3 and 24 h after LPS in comparison with Sal (3 h). Magnitude of low (LF, **e**) and high frequency (HF, **f**) components of PI and LF component of SAP (**g**). *P < 0.05 of LPS-treated group (1.5 and 2.5 mg kg^−1^) compared with Sal, #*P* < 0.05 compared with time zero from LPS-treated group of 1.5 and 2.5 mg kg^−1^. No differenced *P* > 0.05. were observed between LPS doses tested

Administration of both LPS doses (1.5 or 2.5 mg kg^−1^) caused biphasic hypotension (*P* < 0.001, Fig. 2a) and tachycardia throughout the experimental period (*P* < 0.001, Fig. 2b). These cardiovascular responses were independent of the LPS doses used and for these reasons, rats received 1.5 mg kg^−1^ of LPS or Sal in the next experimental protocols.

To verify whether the drop in MAP and tachycardia persist 24 h after LPS was given, we evaluated rats on the day after the treatment, and observed that MAP values reduced at 3 h, but returned to nadir levels in (24 h) group (*P* > 0.05, Fig. 2c). On the other hand, HR remained significantly increased in LPS (24 h) in relation to Sal rats (*P* < 0.05, Fig. 2d). To provide mechanistic insights involved in autonomic control of heart and resistance vessels, spectral analysis of PI and SAP was done and data are showing that LF component of PI was not affected by SI (*P* > 0.05, Fig. 2e). On the other hand, HF component of PI reached lower values at 3 and 24 h after LPS administration in relation to Sal group (*P* < 0.05, Fig. 2f), suggesting a reduction of parasympathetic drive to the heart. LF of SAP was significantly increased in LPS and LPS (24 h) rats than in Sal group, suggesting an increase in sympathetic vasomotor tone after LPS (*P* < 0.05, Fig. 2g).

**Fig. 3:**
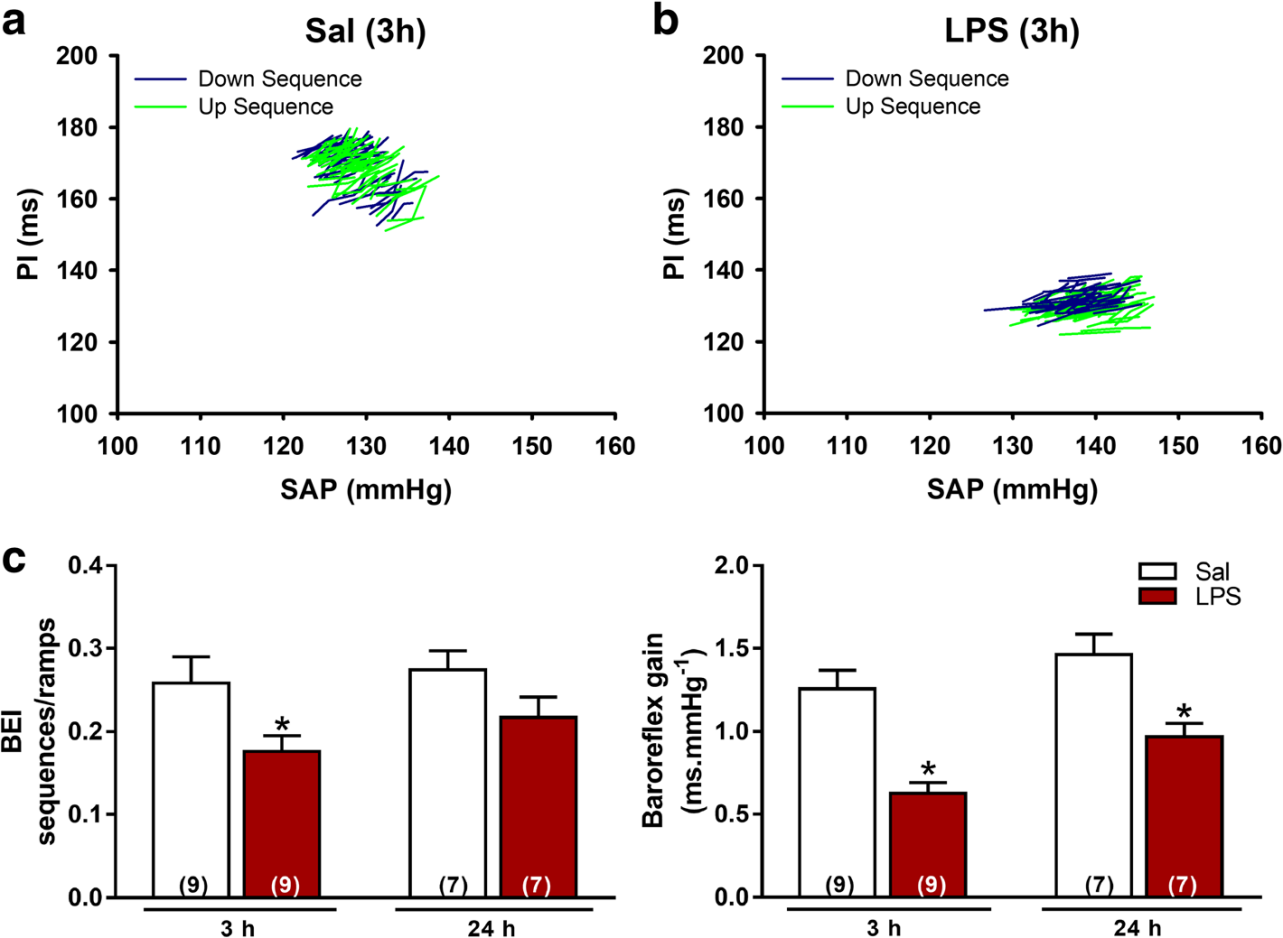
Sequences of spontaneous cardiac baroreflex sensitivity of a representative animal from Sal (**a**) and LPS groups (**b**), 3 h after LPS or Sal administration. Average values of baroreflex effectiveness index (BEI), and baroreflex gain (**c**) after Sal (3 h) or LPS (3 and 24 h). *P < 0.05 compared with Sal

Spontaneous baroreflex gain was significantly reduced in LPS and LPS (24 h) than in Sal group (*P* < 0.05, Fig. 3c). Moreover, the baroreflex sensitivity index (BEI: ratio between sequences and ramps), evaluated 3 h after LPS or Sal injection, was significantly reduced in LPS in comparison to Sal rats (*P* < 0.05, Fig. 3c).

**Fig. 4:**
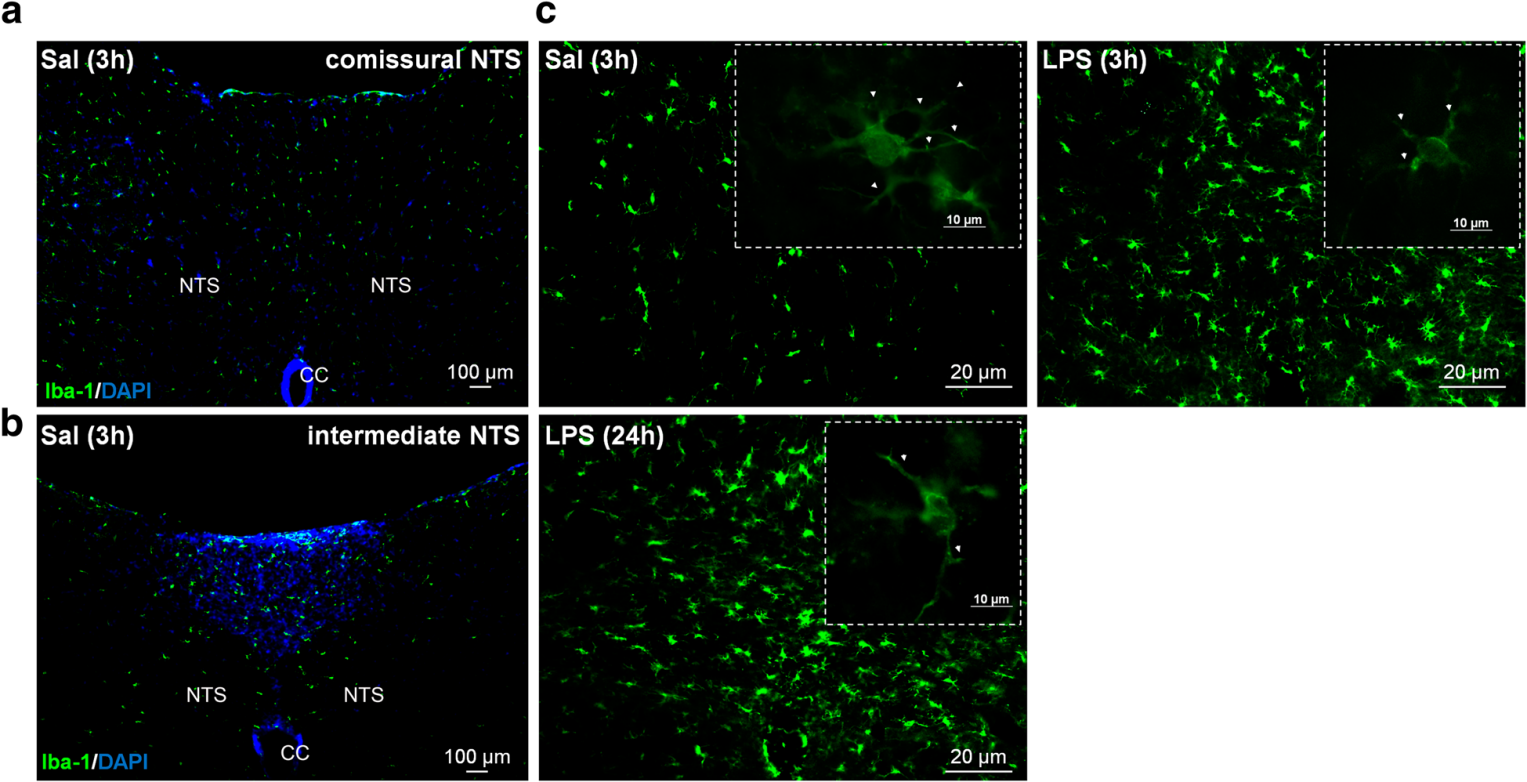
Photomicrographs illustrating Iba-1 microglial marker immunostaining (green) and cell nuclei stained with DAPI (blue) from commissural and intermediate NTS (**a**, **b**, magnification × 2.5). High magnification from Sal (3 h), LPS (3 h), and LPS (24 h) groups (**c**) showing the number of microglia cells in the intermediate NTS region (magnification × 10). The inset on the upper right is showing microglial morphology in the intermediate NTS (**c**, magnification × 20). Central canal (CC)

**Fig. 5:**
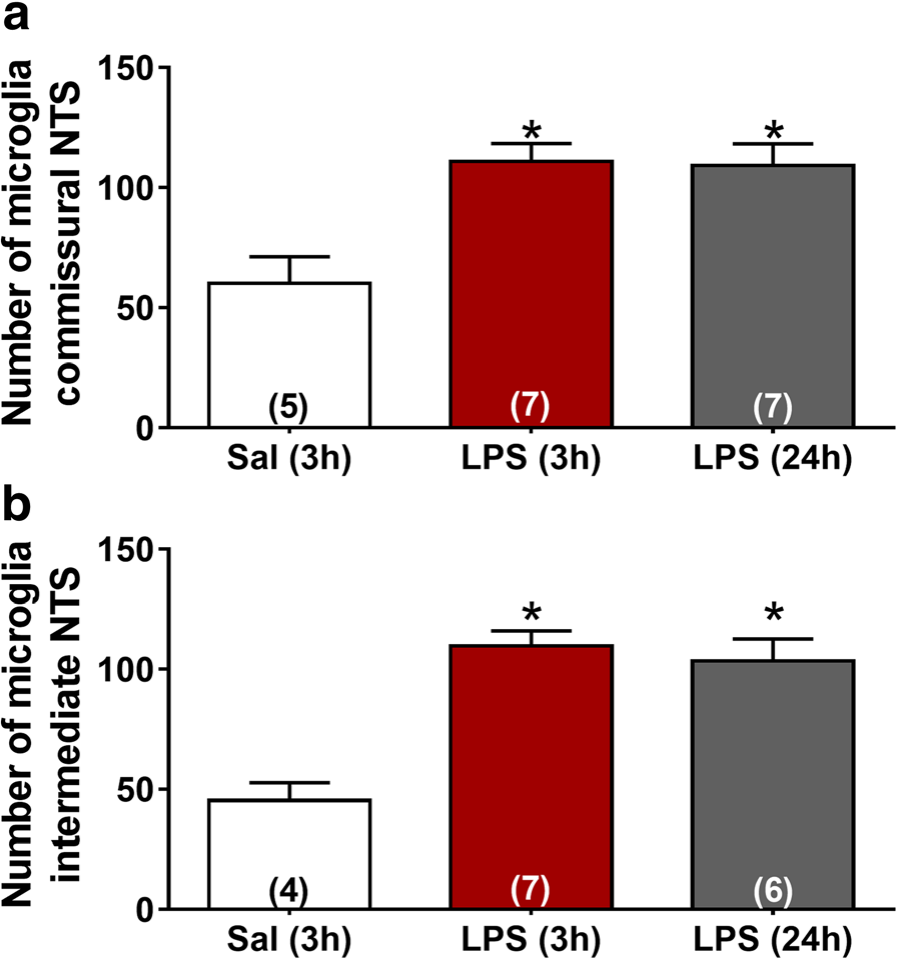
Average values of microglial number from commissural (**a**) and intermediate NTS (**b**) of each experimental group. *P < 0.05 compared with Sal (3 h)

**Fig. 6:**
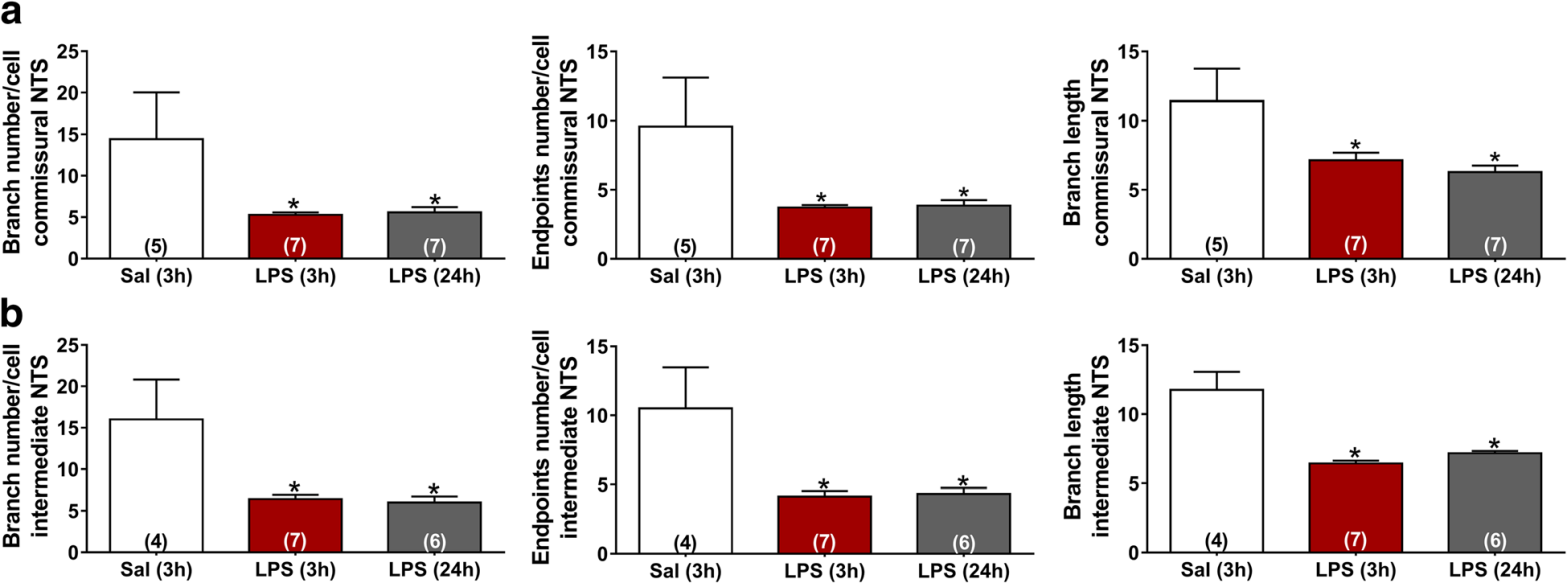
Average of the number of branch, end points, and branch length in the commissural (**a**) and intermediate NTS (**b**) of each experimental group. *P < 0.05 compared with Sal (3 h)

**Fig. 7:**
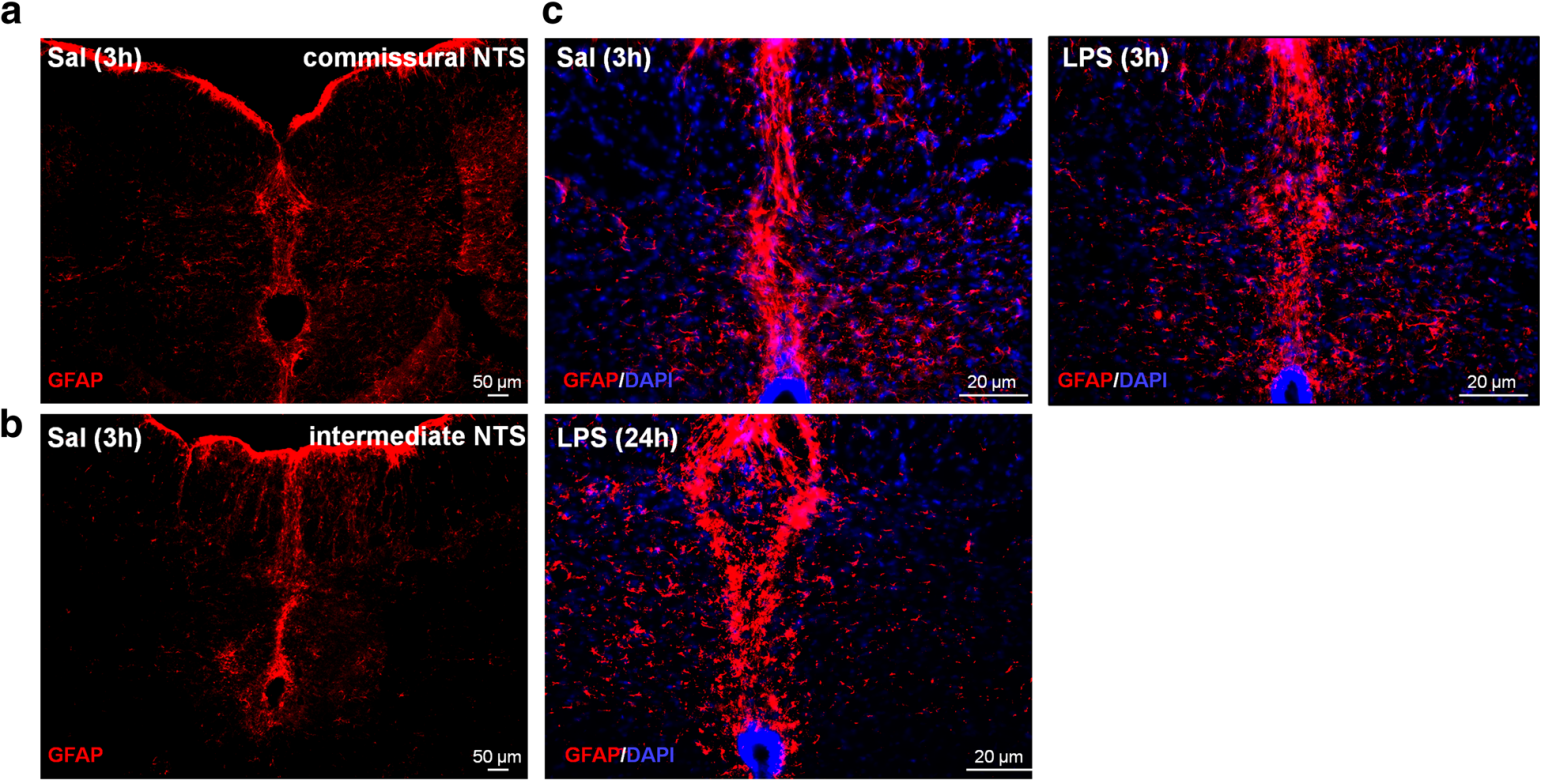
Immunoreactivity of GFAP (red) and DAPI (blue) in coronal sections along the commissural (**a**) and intermediate NTS (**b**). High magnification from Sal (3 h), LPS (3 h), and LPS (24 h) groups (**c**) showing no astrocytes activation in the intermediate NTS region

**Fig. 8:**
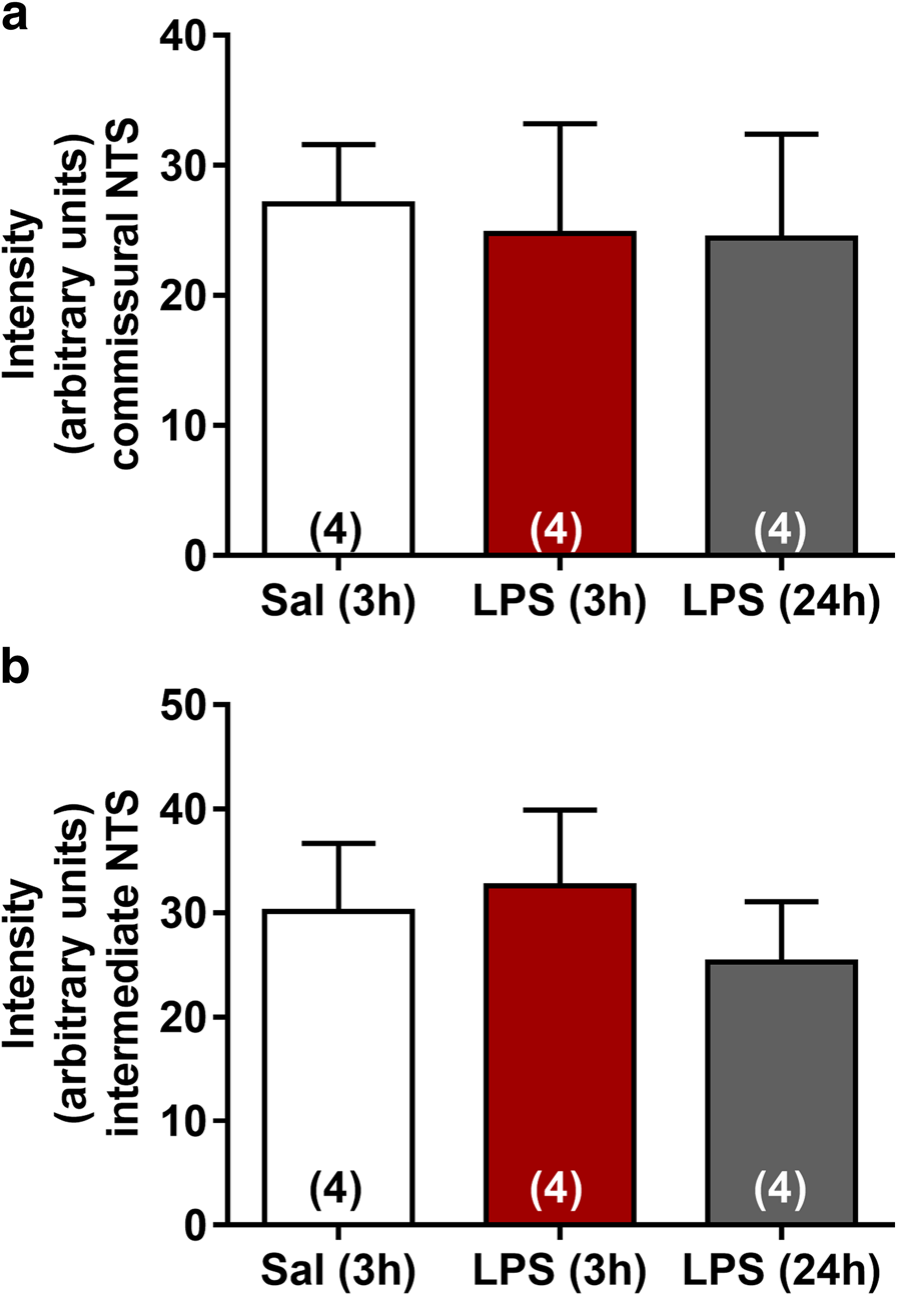
Average values of immunoreactivity intensity within commissural (**a**) and intermediate NTS (**b**) of each experimental group

**Fig. 9:**
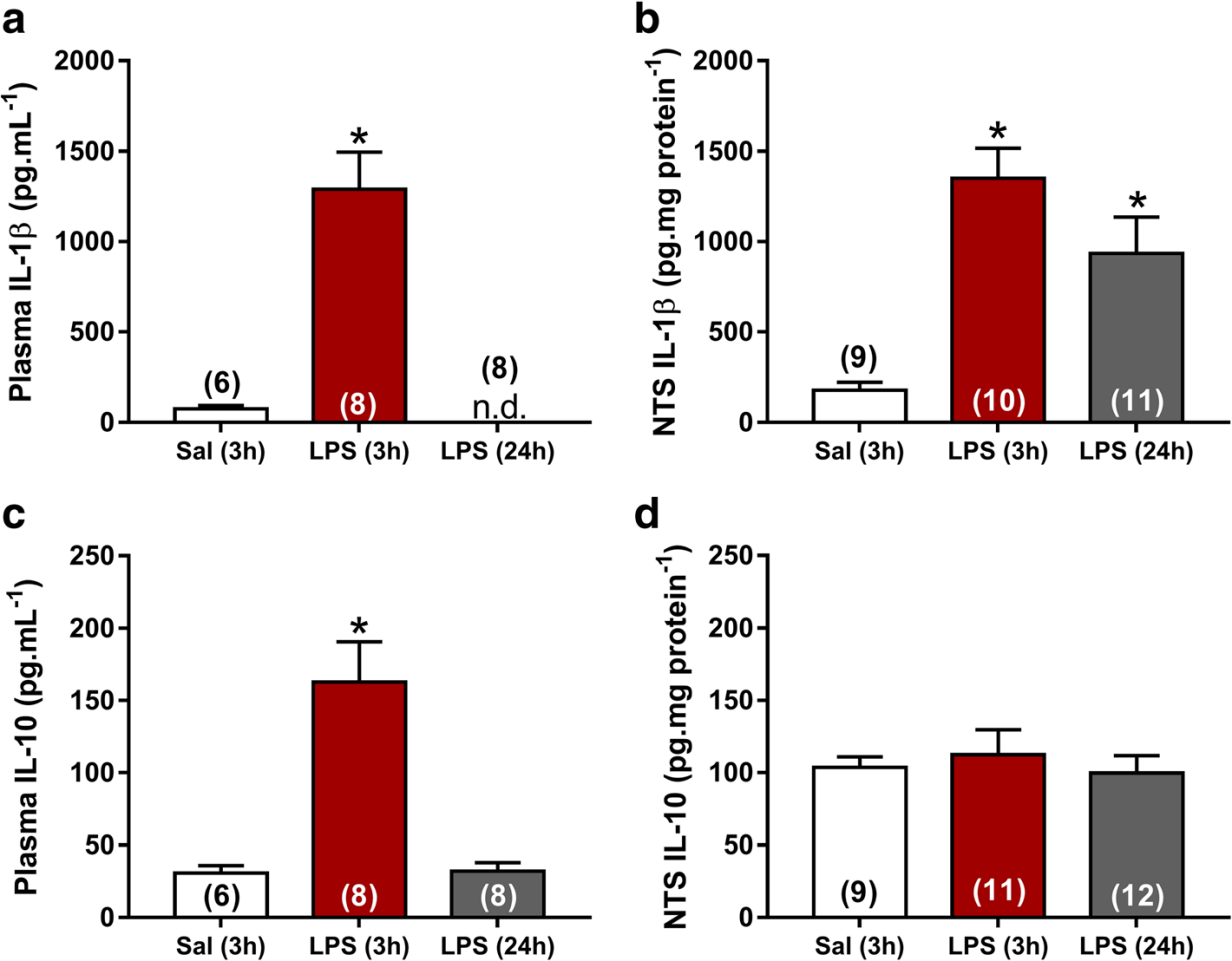
Plasma (**a**, **c**) and NTS (**b**, **d**) levels of IL-1β and IL-10 respectively from Sal (3 h), LPS (3 h), and LPS (24 h) groups. *P < 0.05 compared with Sal (3 h)

NTS is the primary site of cardiovascular and inflammatory afferent terminals [21, 28]. In order to investigate neuroinflammation in the central nervous system (CNS), the number and morphology of microglia were evaluated in commissural and intermediate NTS given that peripheral chemoreceptors and baroreceptor afferents establish synaptic contacts in these regions, respectively. The number of microglia was significantly higher in rats at 3 and 24 h after LPS administration in comparison with the Sal group (*P* < 0.05). This increase was observed in both regions of NTS (Figs. 4 and 5). In addition to changes in the number of microglia during SI, significant changes in the morphology of the Iba-1-labeled cells were also observed (Fig. 6). Microglia from Sal animals were ramified, with long thin processes, whereas during SI, microglia displayed an enhanced hypertrophism and soma enlargement coincident with the activated nature [47]. The number of branches per cell, the branches length, and the endpoints were significantly lower in LPS and LPS (24 h) in relation to Sal group (*P* < 0.05) in commissural and intermediate NTS. Altogether, these observations indicate that microglial cells in NTS are activated at 3 h after LPS administration and that this activation is not reversed 24 h after endotoxemia. Astrocyte activation (GFAP fluorescence) was also quantified herein by measuring the average mean fluorescence intensity in NTS. Representative images from selected points are shown in Fig. 7. The proportion of activated astrocytes did not change among groups (*P* > 0.05, Fig. 8). Peripheral and central IL-1β and IL-10 levels were determined during SI as a useful indicator of the inflammatory status. Acutely, SI increased the IL-1β and IL-10 plasma levels in comparison with Sal rats (*P* < 0.05, Fig. 9a, c). Moreover, 24 h after SI, plasma levels of both IL-1β and IL-10 were reduced to nadir levels. These results indicate that SI-induced plasma IL-1β and IL-10 surges were reversed 24 h after endotoxin administration. A significant increase in IL-1β, but not of IL-10 levels was also observed in the NTS of LPS and LPS (24 h) than in Sal groups (Fig. 9b, d). These findings indicate that SI-induced IL-1β surges in the NTS were not reversed over the time. Considering the clear enhancement in HR after SI, we evaluated the possible correlation between LPS-induced changes in HR and the levels of cytokines. No correlation was found between IL-1β and IL-10 levels and LPS-induced tachycardia (*P* > 0.05).

**Fig. 10:**
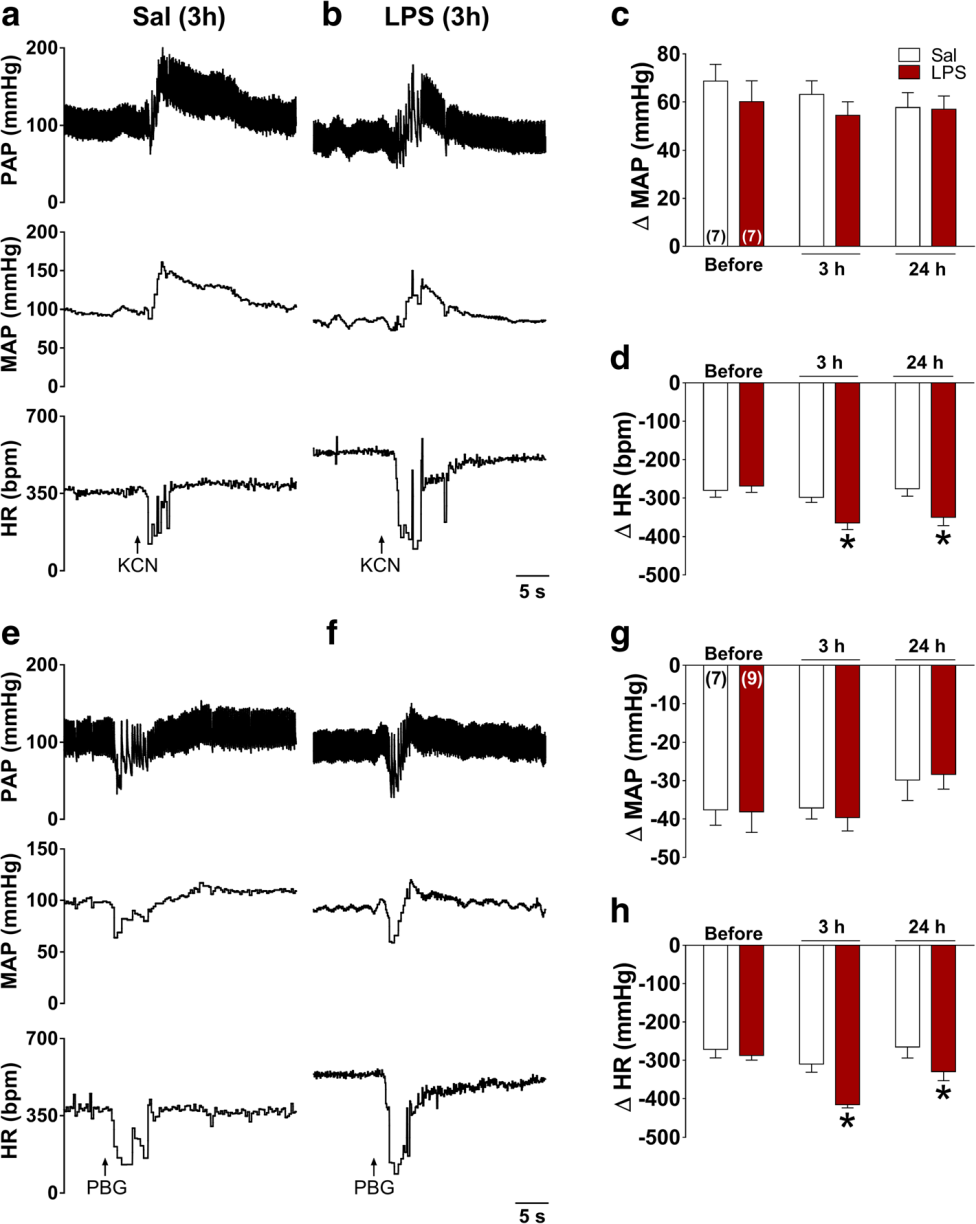
Recordings of PAP, MAP, and HR responses to peripheral chemoreflex activation with i.v. injection of potassium cyanide (KCN, **a**, **b**) and phenylbiguanide (PBG, **e**, **f**) in one representative Sal (3 h left) and one LPS (3 h) rat (middle). Average values of changes in baseline MAP and HR at 3 and 24 h after LPS administration in response to KCN (**c**, **d**) and PBG administration (**g**, **h**)**.** *P < 0.05 compared with Sal

Peripheral chemoreflex receptors located mainly in the carotid bodies are polymodal structures involved in detecting inflammatory stimuli [3, 48]. We tested the impact of SI on hemodynamic responses to pharmacological activation of the peripheral chemoreflex 3 and 24 h after endotoxin administration. Figure 10 shows representative tracings of cardiovascular changes in response to KCN in one Sal rat (a) and in one LPS rat (b). The typical pressor response to chemoreflex activation was not reduced 3 and 24 h after LPS administration (*P* > 0.05, Fig. 10c). Differently to what we observed in MAP, bradycardic component of chemoreflex was significantly higher in LPS than in Sal animals at 3 and 24 h after LPS administration (*P* < 0.05, Fig. 10d).

Bezold–Jarisch reflex was activated by iv administration of PBG and typical changes in MAP and HR are presented in the Fig. 10e. Note that PBG administration induced bradycardia and hypotension in both Sal and in LPS groups (Fig. 10e, f). In addition, LPS group showed a similar hypotensive response to reflex activation compared to Sal group at 3 and 24 h after LPS or Sal administration (Fig. 10g; *P* > 0.05). In spite of similar hypotensive responses to PBG during SI, bradycardic component of the Bezold–Jarisch reflex activation was significantly higher in LPS compared to Sal group at 3 and 24 h after LPS or Sal administration (*P* < 0.05; Fig. 10h).

**Fig. 11:**
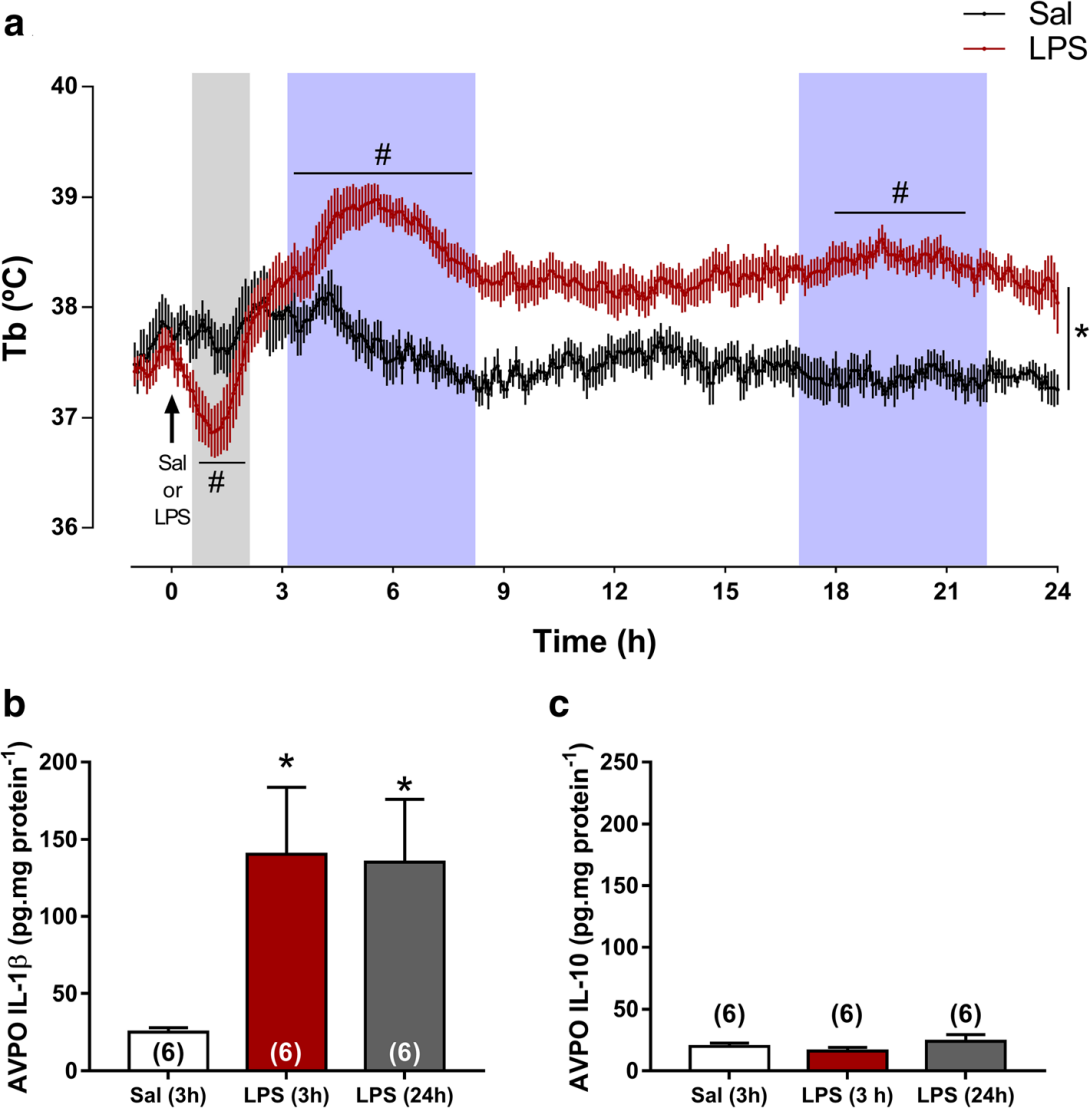
Changes in deep body temperature (Tb) in response to injection of saline (Sal) or LPS (**a**). AVPO levels of IL-1β (**b**) and IL-10 (**c**). *P < 0.05 compared with Sal (3 h), #*P* < 0.05 compared with time zero. The shaded area in gray shows the hypothermia and the blue area shows the fever

Acute and long-term changes in deep body temperature (Tb) were evaluated simultaneously to cardiovascular recordings, to further provide eventual associations between cardiovascular complications during SI and changes in febrigenic signaling. In this set of experiments, typical hypothermia flowed by febrile response was evident at 3 and 24 h after endotoxin administration (Fig. 11). Regarding inflammatory status of AVPO, the most important area of thermoregulation [14] we observed that SI-induced surges of IL-1β, but not of IL-10 levels was observed in AVPO of LPS and LPS (24 h) than in Sal groups (*P* < 0.05, Fig. 11b, c).

## Discussion

The main findings obtained in the present study was the clear-cut demonstration that the bradycardic component of the chemoreflex and Bezold-Jarisch reflex (key regulators of cardiovascular homeostasis) [49] was increased both acutely (3 h) and 24 h following SI. By combining different experiments, the present study is seminal and increases our understanding about hemodynamic, inflammatory, and fibrigenic regulation during neuroinflammation and SI that resembles pathophysiological complications of sepsis [4, 35]. These findings are in line with evidences documenting that electrical stimulation of the cardiovascular reflexes afferents attenuates inflammation during SI [25, 31]. To our knowledge, this is the first study showing the role of cardiovascular reflex control (combining baroreflex, chemoreflex, and Bezold-Jarisch reflex) during SI. Furthermore, these changes in cardiovascular reflexes during SI were associated with neuroinflammation in the NTS.

### Hemodynamic control during SI

The isolated effect of SI in the cardiovascular system of patients with sepsis is difficult to study due to a large variety of related factors [4]. In early stages of sepsis, hypotension takes place due to a plethora of factors such as exacerbated release of vasodilators, especially nitric oxide; impairment of endothelial cells regulatory functions; and reduction in adrenergic sensitivity in the microcirculation [50]. Administration of high doses of LPS, as used in the present study, causes pathophysiological changes similar to that observed during sepsis, such as hypotension, tachycardia, and hypothermia followed by fever [35]. LPS stimulates the innate immune system, interacting with CD14 receptor and Toll-like receptor 4 (TLR4) [11] triggering intracellular signaling cascades that culminate with pro-inflammatory cytokines surges [13] and microglial activation in the CNS [51].

During SI in rats and humans, significant hypotension takes place [35] even in the presence of sympathetic overactivity [52]. Several studies have provided compelling evidence for mechanisms involved in cardiovascular perturbations during SI [53, 54]. The present study also shows that intravenous injection of LPS in a nonlethal dose caused hypotension and tachycardia (Fig. 2). Hypotension leads to an inadequate blood flow and oxygen supply to organs and tissues [55] and must be controlled in septic patients. Tachycardia in turn has been reported as a reflex compensatory response of hypotension [56]. Of particular importance, we observed that 24 h after LPS administration, MAP returned to nadir levels (Fig. 2). On the other hand, LPS-induced tachycardia was kept constant 24 h after SI. Significant changes in the autonomic control to the heart have been observed during the initial phase of sepsis-associated SI as compensatory adjustment lessens circulatory shock [57]. To investigate the mechanistic insights involved in the autonomic alterations to the heart and resistance vessels 3 and 24 h after LPS administration, we performed spectral analysis of PI and SAP that documented a significant reduction in the HF component of PI during SI of both 3 and 24 h, which is in line with the concept that low parasympathetic, but not cardiac sympathetic overactivity causes tachycardia in this experimental model. On the other hand, the LF component of SAP was higher in conscious rats after LPS administration when compared with Sal group, indicating a significant increase in sympathetic activity to the resistance vessels which is insufficient to compensate acute hypotension during SI (Fig. 2).

Another important finding was obtained in the present study using the sequence method to evaluate cardiac baroreflex function in the experimental groups of the present study. This analysis has been intensively used in clinical studies [58]. Our data indicate a significant reduction in the spontaneous baroreflex sensitivity in rats during SI and this dysfunction in the baroreflex was not reversed 24 h after LPS administration (Fig. 3). Regarding BEI, defined as the ratio between the number of all spontaneous SAP ramps and the baroreflex sequences, during SI, rats had a significant decrease in this parameter in comparison with Sal animals evaluated at 3 h after LPS administration. A reduced BEI has been considered as an important factor associated with high mortality risk during cardiovascular diseases [59] and now we are providing evidence that the effectiveness of baroreflex control of the heart may be associated with the critical hemodynamic complications observed during SI. It is also important to mention that baroreceptors afferents are found inside the radial vascular wall of carotid and aorta arteries [60], and that the reduced levels of MAP acutely (3 h) may be related to changes in the carotid sinus pressure, impairment of NTS activation, and relay function due to neuroinflammation during SI. On the other hand, baroreflex gain at 24 h following LPS administration was still decreased despite the normalized MAP, which is in agreement with the concept that during SI, the arterial baroreceptor function is reduced due to a myriad of factors.

### Microglial and astrocytes activation in NTS during SI

The first synaptic contact of cardiovascular reflex afferents is in the NTS, an integrative sensory relay of a variety of physiological functions [28, 61]. We hypothesize that during LPS-induced SI, NTS is shifted toward a pro-inflammatory state. For this propose, we assessed the number and morphological features of microglial cells, the first line of defense in the SNC, as well as the astrocytes activation, the most frequent glial cells in LPS-treated rats. SI caused a significant increase in the number of microglia in both intermediate and commissural NTS (Figs. 4 and 5). In addition, during SI, microglial cell morphology was altered to an activated state with an amoeboid nature and shorter or no processes [62]. Remarkably, increased microglial number and activation were not reversed 24 h after endotoxin administration (Figs. 4 and 6). Interestingly, no changes in astrocytes in the NTS were observed during SI, suggesting that microglia, but not astrocytes, contribute to the neuroinflammation, at least in NTS (Figs. 7 and 8). These findings are in accordance with a previous study that observed that LPS iv injection leads to microglia activation with no changes in either morphology or astrocyte number in the substantia nigra [63] Considering previous study documenting that (i) LPS treatment tended to increase the number of GFAP^+^ astrocytes, but did not changed the number of Iba1^+^ cells in prefrontal cortex and striatum [47] and (ii) our own data from NTS, we suggest that the LPS-induced astrocytic and microglial responses are dependent on the brain area, the LPS dose used, as well as the time-course after endotoxin administration. Previous studies documented that LPS significantly increases c-fos protein expression in the NTS [64]. Whether or not the interplay between microglia, astrocyte, and neurons contributes to the inflammatory modulation into the brainstem [61] is an interesting matter that deserves further investigation.

### Plasma and NTS levels of IL-1β and IL-10 during SI

We observed that IL-1β and IL-10 levels in the plasma were increased in response to LPS, characterizing the experimental model of SI (Fig. 9). It is important to point out that plasma levels of the pro-inflammatory cytokine IL-1β and of the anti-inflammatory cytokine IL-10 returned to control levels 24 h after the endotoxin administration, indicating that this experimental model allows the evaluation of the mechanisms associated with temporal effects of SI. In addition to the SI, a significant increase of IL-1β, but not of IL-10 in NTS, was observed, indicating that LPS administration leads to pro-inflammatory status in NTS. Differently to what we observed in plasma, IL-1β levels in NTS did not return to nadir levels 24 h after LPS (Fig. 9). LPS-induced surges of IL-1β were accompanied by significant microglial activation in NTS. It has been suggested that the combination between increased IL-1β expression in CNS and microglial activation may be useful in the indication of pathogenesis in brain [65]. Another important aspect that may be considered in the interpretation of our findings is that LPS-induced SI can reach other brainstem regions such as area postrema and the subjacent dorsal motor nucleus [66]. Interestingly, in the present study, we observed an association between neuroinflammation in the NTS with changes in cardiovascular reflexes during SI. NTS evaluation was chosen given that the vagal sensory nerve endings are located in this important site in the medulla oblongata. Future studies are, therefore, necessary to identify possible cardiovascular changes induced by neuroinflammation in other central regions.

### Hemodynamic responses to peripheral chemoreflex and Bezold-Jarisch reflex activation during SI

SI caused no impairment in the expected pressor response to chemoreflex activation, either acutely or 24 h after endotoxin exposure (Fig. 10). It is possible that the baseline sympathetic overactivity is not sufficient to compensate hypotension throughout SI, but it may be useful to induce a pressor response during chemoreflex activation. On the other hand, bradycardic component of the chemoreflex activation was significantly higher during SI, indicating that this mechanism of control of HR is not reduced as documented in shock induced by cecal ligation and perforation [67]. This observation is in line with the hypothesis that even when the baseline parasympathetic drive to the heart is reduced during SI (Fig. 2), it may be recruited during chemoreceptor activation probably due to a decrease in the perfusion of the carotid bodies caused by LPS-induced hypotension. Taking into account that (i) bradycardic component elicited by chemoreflex activation was increased and (ii) that the electrical stimulation of the carotid sinus nerve reduced LPS-induced inflammation [25], we speculate that peripheral chemoreceptor activation may represent a new therapeutic strategy in the modulation of inflammatory and cardiovascular signaling during SI.

Reflex control of cardiovascular system also depends on Bezold–Jarisch reflex, that when activated causes hypotension and bradycardia [68]. The present study shows that SI does not reduced the typical hypotensive responses evoked by intravenous injection of PBG. On the other hand, bradycardic response elicited by Bezold-Jarisch activation was significantly increased during SI (Fig. 10). Taking into account that the depressor response induced by Bezold-Jarisch activation is dependent on the bradycardic response [69], it is possible that the cardiovagal component to this reflex is increased during SI. Taking into account that Bezold–Jarisch reflex activation leads to a potent parasympatho-excitation and that electrical vagal stimulation produces anti-inflammatory effects, this reflex may be explored in further studies aiming novel mechanistic insights for treating SI. Considering the significant increase in the parasympathetic component of peripheral chemoreflex and Bezold-Jarisch reflex and the neuroinflammation in NTS during SI, one may speculate that there are significant changes in the activity of vagal afferent and efferent pathways strengthening the interaction between the immune system, CNS, and cardiovascular control. The application of the called “Polyvagal perspective” in cardiovascular changes during SI deserves investigation [70].

Altogether, these findings provide insights on the mechanisms regulating the cardiovascular system during SI and indicate that the improvement of theses reflexes responsiveness can be useful for the management of sepsis.

### Acute and long-term changes in Tb induced by SI

To characterize whether the acute and long-term changes in the cardiovascular reflexes occur simultaneously with the classic changes in Tb during SI, we measured Tb throughout the 24-h period following LPS or Sal administration (Fig. 11). As expected, hypothermia was observed during SI followed by a febrile response [35]. Neuroinflammation in the AVPO was observed at 3 and 24 h after LPS administration marked by an increase in IL-1β. Interestingly, fever and IL-1β surges in AVPO were kept 24 h after LPS administration. Considering that fever tends to have a beneficial impact on immune system and consequently on the survival [71] and that we observed an enhanced bradycardic component of peripheral chemoreflex and Bezold-Jarisch reflex, we speculate that the febrile response and the changes in cardiovascular reflexes are side-events during multiple and complex neurovegetative adaptations associated to SI.

## Conclusions

**Fig. 12:**
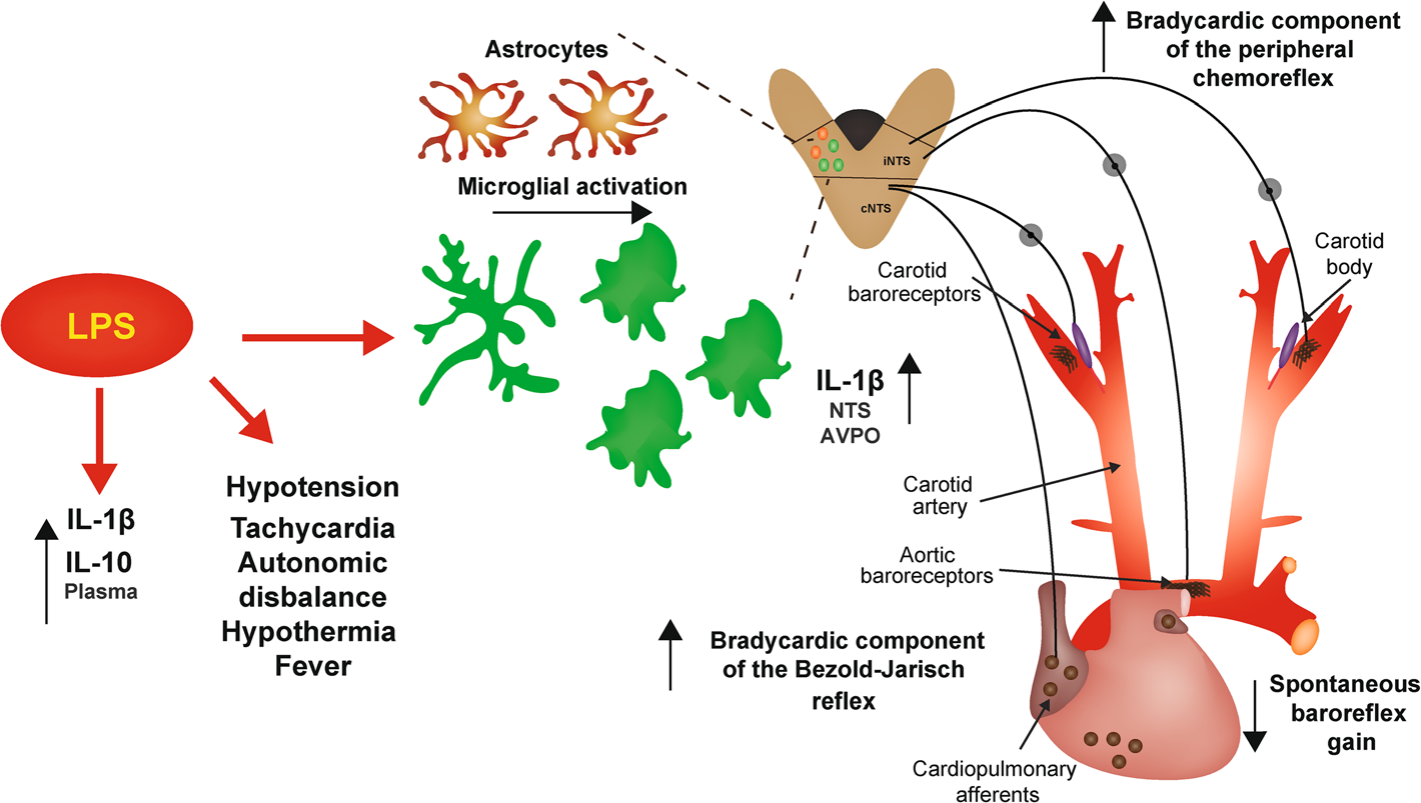
Graphical abstract. Representative diagram with findings of the present study observed after lipopolysaccharide (LPS) administration. Interleukin (IL), nucleus tractus solitarius (NTS), and anteroventral preoptic region of the hypothalamus (AVPO)

This study is the first to show the acute and long-term role of baroreflex, peripheral chemoreflex, and Bezold-Jarisch reflex in the modulation of hemodynamic changes, microglial activation, and IL-1β surges in NTS in the widely used model of SI [10, 18, 64]. In addition, our data added a new dimension that bradycardic component of cardiovascular reflexes (Bezold-Jarisch and chemoreflex) are increased acutely and 24 h following neuroinflammation and SI (Fig. 12). Reconciling the mechanisms associating SI and cardiovascular reflexes is not an easy task; however, we must consider the existent evidence that during neuroinflammation in the NTS induced by hypoxia exposure [61], the sympatho-inhibitory and bradycardiac components of baroreflex and the sympathetic responses of peripheral chemoreflex to activation are increased [72]. For these reasons, a precise modulation of cardiovascular reflexes activity may be a promising therapeutic target during sepsis by increasing the autonomic control of the cardiovascular system and at the same time stimulating the efferents of inflammatory reflex during neuroinflammation in the NTS induced by SI. Therefore, the present findings associated with the existing knowledge open new and interesting perspectives in the field that specific stimulation of both the peripheral chemoreflex and Bezold-Jarisch reflex efferents may represent an advantageous strategy in the management of critical hemodynamic complications of SI.

## Abbreviations

AVPO: Anteroventral preoptic region of the hypothalamus
BEI: Baroreflex effectiveness index
BRS: Spontaneous baroreflex sensitivity
CNS: Central nervous system
ELISA: Enzyme-linked immunosorbent assay
GFAP: Glial fibrillary acidic protein
HF: High-frequency
HR: Heart rate
Iba-1: Ionized calcium binding adaptor molecule-1
IL: Interleukin
KCN: Potassium cyanide
LF: Low frequency
LPS: Lipopolysaccharide
MAP: Mean arterial pressure
min: Minutes
NTS: Nucleus tractus solitaries
PAP: Pulsatile arterial pressure
PBG: Phenylbiguanide
PBS: Phosphate-buffered saline
PFA: Paraformaldehyde
PI: Pulse interval
SAP: Systolic arterial pressure
SI: Systemic inflammation
Tb: Deep body temperature
TLR4: Toll-like receptor 4
